# A comparison of risk and protective factors related to suicide ideation among residents and specialists in academic medicine

**DOI:** 10.1186/1471-2458-14-271

**Published:** 2014-03-22

**Authors:** Mari Eneroth, Marie Gustafsson Sendén, Lise T Løvseth, Karin Schenck-Gustafsson, Ann Fridner

**Affiliations:** 1 Department of Psychology, Stockholm University, Stockholm, Sweden; 2 Centre of Gender Medicine, Department of Medicine, Karolinska Institutet, Stockholm, Sweden; 3 Department of Research and Development, St. Olavs University Hospital, Trondheim, Norway

**Keywords:** Suicide ideation, Academic medicine, Residents, Specialists, Work environment

## Abstract

**Background:**

Physicians have an elevated risk of experiencing suicidal thoughts, which might be due to work-related factors. However, the hierarchical work positions as well as work-related health differ among resident and specialist physicians. As such, the correlates of suicide ideation may also vary between these two groups.

**Methods:**

In the present study, work- and health-related factors and their association with suicidal thoughts among residents (*n* = 234) and specialists (*n* = 813) working at a university hospital were examined using cross-sectional data.

**Results:**

Logistic regression analysis showed that having supportive meetings was associated with a lower level of suicide ideation among specialists (*OR* = 0.68, *95*% *CI*: 0.50-0.94), while an empowering leadership was related to a lower level of suicide ideation among residents (*OR* = 0.55, *95*% *CI*: 0.32-0.94). Having been harassed at work was associated with suicidal ideation among specialists (*OR* = 2.26, *95*% *CI*: 1.31-3.91). In addition, sickness presenteeism and work disengagement were associated with suicide ideation in both groups of physicians.

**Conclusions:**

These findings suggest that different workplace interventions are needed to prevent suicide ideation in residents and specialists.

## Background

Suicidal thoughts are believed to be part of a set of suicidal behaviours that are most serious when they are associated with suicidal plans and actions. Suicidal thoughts thus represent one stage of a continuum that begins with low mood, progresses towards passive death wishes, suicidal thoughts and finally suicidal actions [1-3]. Among individuals who harbour suicidal plans, the probability of actually undertaking a suicide attempt was, according to Scocco (2008), nearly 50 percent [3].

The healthy worker effect refers to the consistent tendency of the actively employed to have a lower mortality rate than the population at large due to selection effects [4]. However, this does not apply to physicians when it comes to death by suicide. Compared with other occupations [5,6], other university graduates [7] and people in general [8,9], suicide and suicidal thoughts are significantly more common among physicians. The mortality rate by all causes of death *except* suicide is however lower for physicians than for the general public [10], which is in line with the healthy worker effect.

To prevent suicides among physicians, it is essential to identify potential risk for, and protective factors against, suicidal thoughts. The present study focuses on the association between work-and health-related factors and suicide ideation among resident and specialist university physicians. The difference between these work positions involves specialists having greater responsibility at work than residents, whereas the work of residents is inherently of a more dependent nature. This hierarchical difference between the two groups makes separate analysis of work-related correlates of suicide ideation necessary.

Among physicians who commit suicide, cases associated with the recent death of a friend or family member or recent crises are less common than for the general public [11]. Instead, it is more likely among physicians for a problem at work to have contributed to the suicide, as was shown in a study concerning the details of suicide from 17 different states in the USA during the years 2003-2008 [11]. Time pressure, work environment, communication, career and goal achievement have previously been found to be work-stressors predictive of high levels of dissatisfaction at work for general practitioners [12].

At university hospitals, physicians’ patient-work is entwined with an academic career. Because of this, the work climate at university hospitals is often extremely competitive and demanding of a high degree of commitment [13]. The presence of a competitive work climate may in turn lead to an oppressive kind of interaction amongst colleagues, or even harassment [14,15]. In a study on Italian and Swedish female physicians, harassment at work was found to be one of the variables that had the largest association with suicidal thoughts [16]. The same study also showed that having meetings to discuss demanding experiences at work was associated with a lower risk of suicidal thoughts among both Italian and Swedish female physicians [16]. Harassment was also found to be related to suicidal thoughts among Italian and Swedish male university physicians, but control over one’s own work seemed to decrease the risk of harbouring suicidal thoughts [17]. In line with this, work autonomy has been seen to have positive effects on the wellbeing of physicians in Sweden, Norway and Iceland [18]. Control over one’s own work was furthermore emphasized in a study of Finnish anaesthesiologists at a university hospital. Workers’ ability to make decisions concerning when and how to apply their own skills, along with organisational justice, were the most important determinants of work-related wellbeing [19].

Residency is the period following internship during which a physician undergoes further clinical training, usually in one medical speciality under the supervision of a specialist. A number of studies have indicated that residents’ psychological wellbeing may also be at risk. The level of depressive symptoms moreover seems to increase with time in residency according to a study using the Patient Health Questionnaire [20]. Scores of depressive symptoms increased from 2.38 at baseline (before entering internship) to 6.26 after 12 months of internship Another study found the lifetime prevalence of suicidal thoughts among residents to be 43.1%, with perceived job stress being one of the factors related to the occurrence of suicidal thoughts during the first postgraduate year [21]. Additionally, perceived medical school stress has been shown to be a predictor of postgraduate mental health problems [22]. However, findings regarding this have been somewhat conflicting, as a more recent study showed that *lower* perceived medical school stress was a predictor of a transition from suicidal thoughts to suicidal planning after graduating [23].

Moreover, medical residents who changed their position (that is, residents who became specialists) experienced an increase in job satisfaction [24]. Decreased job insecurity was believed to be an explanation for this, which suggests that career stage can have an influence on wellbeing at work.

Younger age is in itself a risk factor for suicidal ideation [25,26], and residents are as a group relatively young, so age may be an explanatory factor. In keeping with this, psychological distress has been found to be significantly more common among younger doctors and residents than among their older colleagues [27]. Furthermore, not having children or having just one, compared with having more than one child, is a demographic factor that has been found to be related to work distress among younger doctors [28]. However, this has not been associated with suicidal thoughts among specialists [16,17].

Another possible correlate to suicidal thoughts among residents is sickness presenteeism, that is, working even when sick, which has been found to be common among residents [29]. Sickness presenteeism is also a well-known problem among specialists [30], as it has been seen to be a risk factor for health problems among physicians [31], as well as among the general public [32]. Particularly, the risk for mental health problems is larger among those who work despite being sick [32].

Furthermore, emotional distress is reported to affect physicians’ patient work through an increased irritability, inability to concentrate and reduced work capacity [27]. A way of coping with feelings of exhaustion may be by distancing oneself from patients [33]. Work disengagement can be defined as an uncaring, distanced and cynical attitude toward the work itself or the people with whom one works. It has been found to promote demanding and threatening patient behaviour, which can lead to a vicious cycle as the patient-physician relationship deteriorates [33].

According to previous research, part of the explanation for the greater levels of mental illness, suicidal thoughts and suicides among physicians compared with those in the general public could lie in factors related to their work situation [11]. In addition, the work conditions, hierarchical positions and socio-demographics of residents and specialists are inherently different. Accordingly, their work-related health differs, as indicated by previous research [24,27]. At university hospitals, the different work situations of residents and specialists may be of even greater importance owing to the higher level of engagement that is required at these hospitals [13]. Furthermore, a more competitive work climate may most adversely affect the residents, who have a weaker position at such workplaces. As residents represent the younger group of doctors upon whom we as a public are dependent for care in the future, focusing on this group is especially important. However, as they are still at the starting point of their careers, they may also be more susceptible to interventions. It is essential that the work situation of physicians is acceptable so that psychological distress does not hinder them from caring for their patients, as well as for themselves. Unfortunately, there is a lack of studies that compare residents and specialists with regard to work-related correlates of suicide ideation. For proper examination of the work factors that could protect against, or predict, suicidal thoughts among physicians in different hierarchical levels within their profession, there is a need to study these two groups of physicians separately.

The aim of the present study is thus to investigate the possible correlates of suicide ideation among university hospital resident and specialist physicians separately. For this purpose, logistic regression analysis of variables related to suicide ideation will be performed. It is hypothesized that harassment, sickness presenteeism, work disengagement, exhaustion and the need to demonstrate competence are associated with an increased level of suicide ideation. Control of one’s own work, an empowering leadership and the organisational culture, as well as having meetings to discuss demanding experiences at work, are hypothesized to be associated with a lower level of suicidal ideation. We want to investigate if there is variation in these relationships between residents and specialists.

## Methods

The present study used cross-sectional data from physicians working at a university hospital in Sweden. The data collection in 2005 was organised through a web-based survey by the Department of Research and Development at St Olav’s University Hospital, Trondheim, Norway. The study was presented to the physicians as a research program concerning work-related health, organisational culture, career paths and working conditions in university hospitals. Associate professor, psychologist and psychotherapist Ann Fridner, and professor in psychiatry KG Götestam administered the questionnaires. All participants used personal log-in information to enter their responses to the web-based survey anonymously. Participation was voluntary and the act of logging in served as informed consent. The questionnaire was written in English, which can be considered as a nearly congruent language among university hospital physicians in Sweden. Familiarity with the English language is a prerequisite for completion of medical education, since nearly all of the texts in the medical education program are written in English. The questionnaire took 15-25 minutes to complete and contained 116 questions. Four reminders were sent via e-mail, and a paper version was also sent to all of the physicians. Confidentiality was guaranteed and a website was provided that displayed further information. The Regional Ethics Board in Stockholm examined and approved the project (reference number 04-913/2).

### Participants

Physicians were eligible to participate in the HOUPE study if they were permanently employed as physicians and actively working at a university hospital. The current study includes only Swedish physicians, since the questionnaires in Norway and Iceland did not include questions about suicide ideation. The questionnaire in Italy included items about suicide ideation, but respondents were not included in the Italian survey because they were employed at Padua University, which is not a university hospital.

A total of 1380 specialists i.e. a physician who has undergone residency training, and 447 residents, i.e. a physician in training to become a specialist, were invited to participate in the present study. The response rate among residents was (52%), and (59%) among specialists. There were a greater proportion of non-responders among the male physicians, of whom 56.4% responded, compared with 67.4% of the female physicians. Additionally, there were a greater proportion of non-responders among the male specialists in the age-group of 50-59, among whom only 46.9% responded.

### Measures

Four types of independent factors were measured: work-related factors, health-related behaviour, psychological distress and demographic factors.

### Work-related factors

*The Questionnaire for Psychological and Social Factors at Work* (QPS-Nordic) [34] was used to assess organisational culture (e.g. “Do workers take initiative at your workplace?”) and leadership (e.g. “Does your immediate superior encourage you to participate in important decisions?”), consisting of three items each. QPS-Nordic was also used to assess control consisting of five items (e.g. “Can you influence the amount of work assigned to you?”). The scoring for each of the items ranged from 1 (Very seldom or never) to 5 (Very often or always). The QPS-Nordic was furthermore used to assess the single items of the need to demonstrate competence, which had the same response alternatives as the scales above, and harassment, for which a dichotomous response was possible (yes/no). Harassments were measured with the question: “Have you noticed anyone being subjected to a degrading experience or harassment at your workplace during the last six months?” The question was preluded buy the following definition: “Degrading experiences (harassment, mental bullying, offending somebody) are a problem at some work-places and for some workers. To label something as degrading experiences, the offensive behaviour has to occur repeatedly over a period of time and the person confronted has to experience difficulties defending himself/herself. The internal reliabilities for each scale were 0.74, 0.85, and 0.82 respectively, measured with Cronbach’s alpha. QPS-Nordic has previously been validated and tested for reliability. The test-retest reliability was shown to vary between 0.55 and 0.82 when the interval between the two tests was five to eight weeks [34].

The question: “Are there regular meetings to discuss demanding experiences at work?” was taken from a large-scale research program on physician health and work conditions conducted in Norway [35]. The question had scores ranging from one to three (No; It happens from time to time, but nothing formal; Yes).

### Health-related behaviour

Sickness presenteeism was measured with the question: “Have you gone to work with an illness in a situation where you would have recommended a patient to stay at home?” with scores ranging from one (Very seldom or never) to five (Very often or always). The question was selected from the *Physician Career Path Questionnaire (PCPQ)*[13].

### Psychological distress

The Mini Oldenburg Burnout Inventory (MOLBI) [36] was used to assess disengagement at work (e.g. “Lately I tend to think less during my work and just execute it mechanically”) and exhaustion (e.g. “Have you recently felt constantly under strain?”) measured with five items. The possible answers ranged from 1 (Totally agree) to 4 (Totally disagree). The internal reliabilities of the scales were 0.68 and 0.70, respectively, measured with Cronbach’s alpha.

### Demographic factors

We controlled for demographic factors such as age, civil status (living with a partner or living alone) and number of children. This was done because it was believed that these factors might affect the relationship between suicide ideation and the work-and health-related variables of primary interest in the present study.

### Dependent factor: suicidal ideation

Recent suicidal thoughts were assessed through questions with dichotomous answers (yes/no) concerning the past 12 months. The questions were: “Have you thought about taking your life?” and “Have you thought about specific ways to take your life?”. The responses to these two questions were combined, with an affirmative response to either of them being considered to indicate the presence of recent suicidal thoughts. In other words, if respondents replied that they had not thought about taking their own lives, but still replied that they had thought about specific ways to take their lives, they were considered to harbour suicidal ideation. The questions were obtained from Meehans (1992) survey *Questions about Suicidal Ideation and Attempted Suicide*[37].

## Results

Table 1 shows significant demographic differences between the residents and the specialists. The majority of the residents were women, whereas the majority of the specialists were men (*χ*^*2*^ = 22.87, *p* <0.001). The residents were also significantly younger (*χ*^*2*^ = 418.02, *p* <0.001) than the specialists, and had fewer children (*χ*^*2*^ = 96.29, *p* <0.001).

**Table 1:** Comparison of demographic characteristics of resident and specialists, lifetime statistics of suicidal ideation and outcome variable (suicide ideation within 12 months)

**Characteristics**		**Residents**	**Specialists**	**p†**
**Number of respondents**		**234**	**813**	
Sex	Male	86 (36.8)	443 (54.5)	<.001
	Female	148 (63.2)	370 (45.5)	
	missing data	0	0	
Age group				<.001
	<40 y	190 (81.2)	108 (13.3)	
	40- < 54 y	43 (18.4)	457 (56.2)	
	≥54 y	1 (0.4)	248 (30.5)	
	missing data	0	0	
Living with a partner	Yes	187 (82.4)	616 (77.5)	.113
	No	40 (17.6)	179 (22.5)	
	missing data	7	18	
Number of children	0	76 (33.5)	116 (14.7)	<.001
	1 or 2	124 (54.6)	397 (50.4)	
	>2	27 (11.9)	275 (34.9)	
	missing data	7	25	
Ever had thoughts of suicide	Yes	75 (33.3)	228 (29.0)	.217
	No	150 (66,7)	558 (71.0)	
	missing data	9	27	
Combined outcome variable: had thoughts of suicide and/or thought of specific ways to commit suicide within the past 12 mo.	Yes	29 (12,8)	103 (13.3)	.850
	No	198 (87.2)	674 (86.7)	
	missing data	7	36	

Values are number and (%) of respondents.

**†**p-values measured by χ^2^-tests.

The outcome variable suicidal ideation was assessed by numerical counts and percentages. *χ*^*2*^ tests were used to identify differences between residents and specialists. There were no significant differences in the combined outcome variable: recent suicidal thoughts and/or recent thoughts of suicidal actions (Table 1).

An analysis of work- and health-related factors was also performed using independent samples t-tests to identify differences in the subjective perceptions among residents and specialists. Table 2 shows that the specialists perceived a greater amount of control over their own work (t,= -7,09, *p* <0.001) than the residents did, while the residents more often felt that it was necessary to demonstrate competence in order to be assigned to attractive tasks or projects than the specialists did (t = 4,24, *p* <0.001). Furthermore, the specialists reported more frequent sickness presenteeism than the residents (t = -3.61, *p* < 0.001).

**Table 2:** Comparison of perceptions of work- and health-related factors

		**Residents**		**Specialists**		
		**Mean**	**SD**	**Mean**	**SD**	**p†**
Work related	Necessary to demonstrate competence	3.27	1.10	2.90	1.19	<.001
	missing data	4		18		
	Leadership	2.91	1.01	2.94	1.10	.764
	missing data	6		25		
	Organizational culture	3.15	.84	3.18	.84	.711
	missing data	5		24		
	Control at work	2.44	.76	2.87	.85	<.001
	missing data	9		30		
	Have been subjected to harassment	No.	(%)	No.	(%)	
	Yes	25	11.0	112	14.4	.184
	No	203	89.0	666	85.6	
	missing data	6		35		
	Regular meetings to discuss demanding experiences	No.	(%)	No.	(%)	
	Yes	103	45.0	296	37.4	.087
	It happens from time to time, but nothing formal	77	33.6	322	40.7	
	No	49	21.4	173	21.9	
	Missing data	5		22		
Health behaviour	Sickness presenteeism	2.75	1.20	3.08	1.18	<.001
	missing data	6		27		
Psychological distress	Disengagement at work	2.18	.50	2.22	.49	.253
	missing data	4		17		
	Exhaustion	2.60	.53	2.59	.55	.694
	missing data	4		17		

Values are mean and SD.

**†**p-values of differences in mean measured by independent samples t-tests and χ^*2*^*-*test.

Logistic regression analysis was performed with each of the independent variables separately. This was done in order to study how the odds ratio (estimated risk) of having experienced recent suicide ideation was affected by the presence or absence of each of the independent variables. Table 3 shows the separately analysed independent variables that were significantly associated with recent suicide ideation. This reveals differences among the two groups, such as that an empowering leadership was associated with a lower level of suicide ideation for residents only (*OR* = 0.44, *95*% *CI*: 0.28-0.69). The perception of a need to demonstrate competence was related to suicide ideation for specialists (*OR* = 1.22, *95*% *CI*: 0.83-1.77). Furthermore, Table 3 shows that having been subjected to harassment was associated with a higher level of suicide ideation among specialists only (*OR* = 3.09, *95*% *CI*: 1.90-5.03).

**Table 3:** Factors with significant (p ≤0.05) non-adjusted odds ratios (ORs) for recent suicidal thoughts among the resident and specialist physicians

**Group**	**Independent variables**	**OR**	**95% CI**	**p**
**Residents**				
Work related factors	Leadership	.44	.28-.69	<.001
	Regular meetings to discuss demanding experiences at work	.55	.31-.97	.040
	Organizational culture	.52	.32-.84	.007
Health related behaviour	Sickness presenteeism	1.46	1.04-2.04	.027
Psychological distress	Disengagement at work	5.91	2.53-13.80	<.001
**Specialists**				
Work related factors	Necessary to demonstrate competence	1.22	.83-1.77	.029
	Have been subjected to harassment	3.09	1.90-5.03	<.001
	Regular meetings to discuss demanding experiences at work	.61	.46-82	.001
	Organizational culture	.76	.60-.98	.031
Health related behaviour	Sickness presenteeism	1.47	1.22-1.77	<.001
Psychological distress	Disengagement at work	3.54	2.30-5.44	<.001

Each of the demographic variables was also analysed in terms of its separate connection to suicide ideation, with results showing that there were no significant associations between age, civil status and suicide ideation. However, having children was associated with a lower level of suicide ideation among residents (*OR* = 0.63, *95*% *CI*: 0.41-0.97, *p* = 0.34).

Finally, multiple logistic regression was performed to rule out variables that were no longer significant in their predictive or protective value due to co-variation, and also to adjust for socio-demographic variables. Table 4 shows that an empowering leadership was associated with a lower level of suicide ideation among residents (*OR* = 0.55, *95*% *CI*: 0.32-0.94), whereas regular meetings to discuss demanding experiences at work were associated with a lower level of suicide ideation among specialists (*OR* = 0.68, *95*% *CI*: 0.50-0.94). It is furthermore shown that having been subjected to harassment was related to a higher level of suicide ideation for specialists (*OR* = 2.26, *95*% *CI*: 1.31-3.91). Sickness presenteeism was associated with a higher level of suicide ideation for both groups, although just at the boundary of significance for residents (*p* = 0.051) (resident *OR* = 1.48, 95% *CI*: 1.00-2.20; specialist *OR* = 1.29, *95*% *CI*: 1.05-1.58). Disengagement at work was a factor highly related to suicide ideation for both groups of physicians (resident *OR* = 2.74, *95*% *CI*: 1.00-7.47; specialist *OR* = 2.90, *95*% *CI*: 1.75-4.80).

**Table 4:** Factors with significant (p ≤0.05) adjusted odds ratios (ORs) for recent suicidal thoughts among the resident and specialist physicians

**Group**	**Independent variables**	**OR**	**95% CI**	**p**
**Residents**				
Work related factors	Leadership	.55	.32-.94	.029
Health related behaviour	Sickness presenteeism	1.48	1.00-2.20	.051
Psychological distress	Disengagement at work	2.74	1.00-7.47	.050
**Specialists**				
Work related factors	Have been subjected to harassment	2.26	1.31-3.91	.003
	Regular meetings to discuss demanding experiences at work	.68	.50-.94	.024
Health related behaviour	Sickness presenteeism	1.29	1.05-1.58	.016
Psychological distress	Disengagement at work	2.90	1.75-4.80	<.001

Multiple logistic regression with adjustment for socio-demographic factors; age, number of children and marital status.

## Discussion

The aim of the current study was to investigate separately possible correlates of suicide ideation among resident and specialist university physicians. Comparisons between the participating resident physicians and specialist physicians revealed significant differences with respect to demographic characteristics and work-related factors. There was however no difference between the two groups in terms of the prevalence of recent suicide ideation.

According to previous research, autonomy at work is an important factor for wellbeing and work-related health [18,19], including suicidal thoughts [17]. However, the findings of the present study do not support the hypothesis that control over one’s own work is associated with a lower level of suicide ideation, as this factor was not related to suicidal thoughts for either of the two groups of physicians. Even though residents perceived significantly less control over their own work than specialists, there was no connection between this variable and suicidal thought. Exhaustion was also not related to suicidal thoughts in the present study. This lack of correlation was unexpected as exhaustion is considered to be one of the core elements of burnout [33], which in turn has been identified as being connected to suicidal thoughts [38].

A positive organisational culture and the need to demonstrate competence were, in the present study, regarded as concepts related to organisational justice, which has previously been shown to be one of the most important determinates of work-related wellbeing [19]. Previous research has furthermore shown that the work climate at university hospitals is highly competitive [13]. However, the hypothesis that a positive organisational culture would be associated with a lower level of suicide ideation among the participating physicians was not supported in the present study. The hypothesis that the need to demonstrate competence would be related to suicide ideation also failed to gain support. As hypothesized in the present study, having been subjected to harassment was associated with a higher level of suicide ideation among specialists. However, this did not apply to residents, for whom there was no association between harassment and suicide ideation. According to previous research, harassment is a major risk factor for suicidal thoughts among physicians [16,17], including medical students [39], as well as for the general public [40]. The association found in the current study between harassment and suicide ideation for specialists alone might suggest that the negative impact of harassment increases with time and/or career advancement. Perhaps this is because competition regarding positions and research funding also increases as one ascends through the hierarchy, as shown by Fridner (2004) [13]. To be involved in research for more than 50% of one’s working hours, which is limited to only a small number of physicians high up in the hierarchy, has also been found to increase the level of harassment experienced [41].

An empowering leadership was associated with a lower level of suicide ideation among residents, but not among specialists. That an empowering leadership is not associated with suicide ideation for specialists could be explained by the differing positions that the two groups of physicians hold. Specialists are more autonomous than residents, as they are more skilled, and are hence not as dependent upon their bosses. The results obtained in this study might thus be understood to support the assertion that different kinds of leadership are needed for different kinds of worker, as suggested in the Hersey and Blanchard model (1969) [42], in which the leadership style matches the maturity and skill of the employees. For example, if the maturity level of the employees is high, the model suggests a delegating style of leadership with minimal guidance from the leader, whereas the leadership of less mature employees should be of a more supportive kind [42]. Assuming that this leadership approach was applied at the current hospital, this could explain why specialists do not seem to benefit from encouraging leadership, while residents do. The result of the present study is encouraging as it indicates that certain important individuals at a workplace can have a major positive impact on residents.

Having frequent meetings to discuss demanding experiences at work was associated with a lower level of suicide ideation among specialists before and after adjusting for demographic variables. But among residents these meetings were not significantly associated with a lower level of suicide ideation when we adjusted for demographics. Previous research shows that the prevalence of frequent meetings to discuss demanding situations at work is associated with a lower level of suicidal thoughts among female physicians [16]. That residents do not seem to benefit from meetings in this respect may entail that they perceive barriers in engaging in discussions about difficulties that they are having at work, something that could possibly be due to job insecurity. To a significantly higher degree than specialists, residents find it necessary to demonstrate competence at work. This might explain why they do not seem to be relieved by talking about demanding experiences. That is, it is possible that residents fear that engaging in a discussion about their difficulties at work could make them seem incompetent. Personal vulnerability and a feeling that the need for supervision and support could indicate incompetence have previously been reported as factors hindering the implementation of successful group-based supervision among psychiatric nurses [43]. In consonance with this are findings showing that group-based clinical supervision is experienced as difficult because of uncomfortable exposure to one’s peers and superiors [44]. Perhaps individual supportive talks would be a more beneficial alternative for residents, as the ones in most need of guidance and support might also be the ones who find it most difficult to discuss problems openly. In other words, residents may feel freer to talk about difficulties with one or a few trusted individuals, instead of an entire work-team.

Sickness presenteeism appeared to induce an elevated risk of suicide ideation in both groups. This result is in consonance with previous research [31,32], demonstrating a connection between sickness presenteeism and poorer health. Working while sick is believed to be part of a larger behavioural pattern where university hospital physicians neglect their own health. Factors associated with a competitive climate and myths about the healthy doctor are assumed to contribute to this [31]. In addition, the current study further demonstrates the highly detrimental correlates of work disengagement, known as one of the core elements of burnout [33], as it seems to be closely related to recent suicide ideation.

The demographic factors of age and civil status have previously internationally been shown to be connected to suicidal thoughts as an older age and being married are protective against suicidal thoughts among the general public [25,26]. Contrary to this, in the present study, no connection was found between age and civil status and suicide ideation. Only number of children had a significant association with recent suicide ideation, and only among residents, for whom the estimated risk of having had recent suicide ideation seemed to be decreased by having children. This finding is in line with previous research showing a connection between fewer children and work distress among younger physicians [28]. It is possible that children have a positive effect on the wellbeing of residents because they are a distraction from the supposedly negative focus on competitive work and intense medical training.

Suicidal ideation was not associated with work control, organisational culture, the need to demonstrate competence and exhaustion. However, harassment at work, having meetings to discuss demanding experiences, empowering leadership, sickness presenteeism and work disengagement were all related to suicidal ideation. The current study further demonstrates that the work-related factors that are most important for the occurrence of recent suicide ideation among physicians largely seem to depend on position. Harassment at work, having meetings to discuss demanding experiences at work and an empowering leadership were related to suicidal ideation in different ways for residents and specialists.

### Limitations

In the present study, the focus has been on differences in suicide ideation on the basis of position; hence, stratification by sex was not included. Sex-stratified multiple logistic regression with adjustment for age, number of children and civil status, however, indicated differences among specialists, such as that harassment was related to suicide ideation only among female specialists. Additionally, sickness presenteeism was related to suicide ideation only among male specialists. Consequently, analysis stratified by sex is called for in continued research on suicide ideation among university hospital physicians. There were, however, no sex differences among residents at this level of analysis.

The study is based on cross-sectional data. The possibility of making causal interpretations of the findings is therefore limited. A potential source of bias could furthermore be suggested in the fact that data on the outcome variable and the work- and health-related variables were all obtained from the same questionnaire. However, this was partly counteracted by the participants not having been informed about which variable was the outcome variable. Additionally, over-reporting of suicidal thoughts among physicians seemed unlikely owing to the stigmatization associated with mental illness [45].

It should also be noted that only working physicians participated in this study. This fact may have biased the results as physicians who had quit their jobs because of ill health, which one would assume could be related to their work situation, were ineligible. In other words, the obtained associations between work- and health-related factors and the outcome variable may have been lower than the true levels as the study selection prevented the inclusion of physicians who might have generated the highest correlations.

Furthermore, the homogeneity of the sample (Swedish physicians working at a university hospital) should be taken into consideration when attempting to generalize the results of the present study. However, as there is a lack of similar studies in different countries, the present one can be used as a comparison in future research on the subject. An additional limitation to the study is that data are from 2005, thus emphasizing the need for further studies in this important field.

## Conclusions

Physicians’ thoughts of suicide and/or thoughts of suicidal actions are alarming as this group may be more aware of effective methods for actually performing these actions than the general population. Establishing which factors are related to suicidal thoughts is central to the process of preventing suicides among physicians. The current study demonstrates that these factors differ depending on one’s employment position within the medical hierarchy, suggesting that, when dealing with work-related distress among physicians, residents and specialists require separate interventions. However, this study should be extended by obtaining longitudinal data on the work-related predictors of suicide ideation among resident and specialist university hospital physicians. Future similar studies are recommended to stratify the subjects by medical speciality.

## Competing interests

There are no competing interests for any of the authors (neither financial nor other).

## Authors’ contributions

AF is the coordinator of the HOUPE study and the principal investigator in Sweden. AF and KSG were responsible for data collection and are guarantors of the study. AF and LTL prepared the datasets. ME, AF and MGS reviewed the literature. ME and AF analysed and interpreted the data, and wrote drafts of the manuscript. MGS, Gustafsson, LTL and KSG revised the manuscript. All authors read and approved the final version of the manuscript.

## Pre-publication history

The pre-publication history for this paper can be accessed here:

http://www.biomedcentral.com/1471-2458/14/271/prepub
